# Improvements in the nutritional quality of US young adults based on food sources and socioeconomic status between 1989–1991 and 2011–2014

**DOI:** 10.1186/s12937-019-0460-4

**Published:** 2019-06-26

**Authors:** Matthew A. Patetta, Lilia S. Pedraza, Barry M. Popkin

**Affiliations:** 1 Department of Nutrition, Gillings School of Global Public Health, Carolina Population Center, CB #8120 Carolina Square, University of North Carolina at Chapel Hill, Chapel Hill, NC 27516-2524 USA; 2 National Institute of Public Health in Mexico, Cuernavaca, Mexico; 3 Cornell University, Ithaca, USA

**Keywords:** Young adults, Diet quality, Nutrition, Socioeconomic disparity, Healthy eating index

## Abstract

**Background:**

Fast food and other away from home food sources are linked with poorer diet quality and adverse health outcomes. The diet quality of young adults, major consumers of fast food, is understudied in terms of long-term shifts based on food sources for key subpopulation disparities.

**Methods:**

The study included young adults ages 18–39 (*n* = 8012) from the Continuing Survey of Food Intakes by Individuals 1989–1991 (*n* = 4217) and the National Health and Nutrition Examination Survey 2011–2012 and 2013–2014 (*n* = 3795). We stratified individuals based on their combination of food sources, race/ethnicity, and socioeconomic status. Using 24-h dietary recall data, we measured diet quality with the Healthy Eating Index-2015 (HEI-2015). Differences in diet quality were determined using 95% confidence intervals.

**Results:**

Overall, diet quality increased across all food sources between the 1989–1991 and 2011–2014 surveys. The restaurant category overtook the at home category as the healthiest food source, while the fast food category remained the unhealthiest on days it was consumed. Vegetable intake decreased, while added sugar intake increased across all sources. Non-Hispanic whites and non-Hispanic blacks experienced similar increases in HEI-2015 scores across all food sources except restaurants, while Mexican American diet quality remained unchanged. Although all income levels experienced an increase in diet quality, the disparity between low- and high-income groups increased considerably.

**Conclusions:**

US young adults consume healthier foods from all food sources, however, fast food consumers have significantly lower quality in the remainder of their diets. Additionally, Mexican Americans and low-income individuals emerge as high-risk groups for poor diet quality.

**Electronic supplementary material:**

The online version of this article (10.1186/s12937-019-0460-4) contains supplementary material, which is available to authorized users.

## Introduction

Over the past few decades the prevalence of young adults ages 18 to 39 affected by obesity has increased dramatically [1–4]. Currently, the prevalence of obesity in US young adults is 34%, while the prevalence of combined overweight or obesity is 66% [2–4]. During this time food consumption from at home sources, that is, foods prepared in the home environment, also decreased [5–9]. Reflecting this, the percentage of energy consumed at home decreased from 92% in 1965 to 68% by 2008 [5]. Calories from away from home sources, such as full-service restaurants and fast food restaurants, increased from 14 to 34% between 1977 and 2006 [7–10]. Although the percentage of energy from away from home sources decreased to 28% by 2009 in response to the Great Recession, recent trends in NHANES data indicate a resurgence of away from home eating, as calories from these sources contributed 32% of the energy in the overall diet in 2014 [9, 11].

These shifts in eating patterns have occurred differentially across race/ethnic and income groups, often widening nutritional disparities between various socioeconomic statuses [12, 13]. For example, from 1999 to 2012 the amount of non-Hispanic white (NHW) adults with poor diets, as defined by the American Heart Association 2020 Strategic Impact Goals, declined significantly from 54 to 43%, while no significant improvements were observed for non-Hispanic black (NHB) or Mexican American adults [12]. Additionally, from 1999 to 2010 the difference in Alternate Healthy Eating Index 2010 scores, a diet quality measure, between high- and low-income individuals increased from 4 to 8, further revealing the widening of this nutritional disparity based on income level [13].

The most apparent problem with this transition toward full-service restaurants and fast food restaurants (hereafter referred to as restaurants and fast food, respectively) is that it is commonly accepted in the literature that the food served at both sources is often of poorer nutritional quality compared to foods prepared at home [10, 14–18]. Away from home sources have been associated with higher levels of 179 kcal per day (kcal/day), 3.5 g/day of saturated fat, and 411 mg/day of sodium [8, 18]. Moreover, fruit and vegetable consumption, as well as vitamin A, carotene, vitamin C, calcium, and magnesium intake, have been negatively correlated with food prepared away from home [15, 19–21]. Additionally, prospective studies have associated away from home food consumption with greater risk of becoming overweight or obese [22, 23]. It is important to note, however, that fast food is often of worse nutritional quality than restaurant food [17, 24, 25].

Some research claims that increased consumption of foods from away from home sources has a significantly smaller effect on people’s health, specifically in weight gain, than previously theorized [26, 27]. For example, one study suggested that the correlation between obesity and both HEI-2005 scores and total caloric intake of away from home food sources is likely to be overstated [26, 27]. Nonetheless, evidence has shown that one reason the obesity epidemic has worsened over the past few years is an increase in total calories consumed and positive energy balances [2, 4, 26–28]. To resolve this discrepancy in the literature, new studies using 24-h dietary recall data from NHANES have examined the relationship between away from home consumption and the quality of the remainder of the diet [16, 20, 29]. These studies have revealed that individuals who consume fast food frequently consume less nutritious foods the remainder of the day, indicated by greater intakes of total calories, saturated fat, sodium, and sugar-sweetened beverages alongside lower intakes of dairy products, vegetables, and fruits [27, 29, 30]. Despite these findings, the evidence is limited, because research has focused only on the correlation between fast food and the remainder of the diet while largely ignoring restaurants. Moreover, these studies did not investigated how differences in diet quality based on the combination of food sources consumed during a 24-h period may have changed over the years, but rather focused solely on a particular point in time [29, 30]. Additionally, young adults, who as a group have shown rapid increases in weight gain and a high proportion of calories from away from home food sources, are relatively understudied in the current literature [1, 31].

To better understand the relationship between different away from home sources and the remainder of an individual’s diet, this study investigated whether the overall diets of consumers of certain food sources vary from those of other groups of consumers by analyzing dietary information of 24-h recalls of young adults (ages 18–39) in 1989–1991 and 2011–2014. The study used the Healthy Eating Index-2015 (HEI-2015) to assess differences in the nutritional quality of foods consumed on a given day across food sources. Additionally, this study determined changes in HEI-2015 scores over time by examining the 23-year change from 1991 to 2014. Finally, we determined any socioeconomic disparities by analyzing differences in HEI scores across various income levels and race/ethnicity subgroups.

## Methods

### Study population

This investigation used data from the Continuing Survey of Food Intakes by Individuals (CSFII) 1989–1991, the National Health and Nutrition Examination Survey (NHANES) 2011–2012, and the NHANES 2013–2014. We combined the two NHANES into one time period to obtain a sample size comparable to the CSFII 1989–1991. Since this investigation used public data, institutional review board approval was not required. The study examined 24-h dietary recall data for 4217 young adults from the CSFII 1989–1991 and 3795 young adults from the NHANES 2011–2014. We chose the CSFII 1989–1991 over other surveys, such as the NHANES II (1976–1980) and the NHANES III (1988–1994), because of the CSFII’s similarities with the NHANES 2011–2014, including data collection through in-person interviews using analogous language; large, well-balanced samples not focused on a particular demographic; and comparable food composition tables [32–34]. One major difference between the CSFII 1989–1991 and the NHANES 2011–2012 and 2013–2014 is that the CSFII did not use a multiple-pass approach, hence some underreporting in the earlier survey might be possible.

We used only the first of the two 24-h dietary recalls of each survey for three main reasons. First, the collection methods for the second day data differed for each survey, preventing comparability. For example, the CSFII 1989–1991 used in-person interviews for the second day, whereas the NHANES 2011–2012 and 2013–2014 used phone interviews [32–34]. Second, we wanted to assess the diet quality of the population associated with food sources on days when such sources were consumed, rather than assess the population’s usual intake by estimating the probability of food source consumption based on multiple days. This could not be done using a second dietary recall, as one day food sources might differ from the other. Finally, we wanted comparability with dietary studies that use data from one 24-h dietary recall [5, 11, 35].

Our investigation considered only two points in time to capture the overarching diet quality changes that occurred in the past two decades. Furthermore, the selected time points used similar food composition table and dietary collection method, allowing comparison. This study includes male and female young adults ages 18 to 39. Further details of the study designs and procedures of the CSFII and the NHANES are published elsewhere [32–34].

### Socioeconomic classification

Part of this investigation addresses the effects of socioeconomic characteristics on diet quality, in particular race/ethnicity and income. In regard to race/ethnicity, we stratified the study population into three categories: NHW, NHB, and Mexican American. We excluded groups such as Native Americans and Asian Americans that made up less than 5% of the sample population in either the CSFII or the NHANES. We designated Mexican Americans rather than all Hispanics because, while the CSFII 1989–1991 survey did recognize other Hispanic groups, it only publicly released data pertaining to Mexican Americans [32]. Thus to maintain comparability among survey subgroups, we included only Mexican Americans from the NHANES population even though the surveys recognized other Hispanic subgroups [33, 34].

We evaluated income based on the annual family income in relation to the federal poverty level (FPL) for that year [36]. The low-income group had incomes below 180% of the FPL, the middle-income group 180–350%, and the high-income group greater than 350%. It is important to clarify, however, that we did not apply exclusions based on race/ethnicity or income information during the overall population analysis. In other words, we only used these specific race/ethnicity and income levels in the subsequent stratified analysis.

### Food classification

For classification we used information on where the food items were prepared but not necessarily consumed. Based on the information collected in the CSFII and the NHANES, we reclassified specific food sources into four main food source categories depending on where the source fit best: at home, restaurant, fast food, or other. We utilized the NIH and NHANES definition for food establishments, where full service restaurant is defined as establishments that sell foods with waiters/waitresses, while fast food sources are defined as establishments that sell foods that are readily available for consumption without utilizing waiters/waitresses [33, 34, 37, 38]. We selected these categories since they were the most commonly reported food sources in the CSFII and NHANES data. We did not use the “other” classification, which was composed of sources that did not fit well into the other main categories, in this study, because sources such as school cafeteria and child care center are irrelevant for the age range of our sample. Moreover, the “other” sources contributed a minor amount (< 5%) of the total calories consumed by the study population.

### Study population stratified by food source

After classifying the food items individuals ate during a 24-h period by food source, we stratified participants into four groups based on the combinations of their food sources during that period. The at home group consisted of individuals who only consumed food from an at home source. The restaurant group included people who ate foods from restaurants and at home sources. The fast food group consisted of participants who ate from fast food and at home sources. Finally, the mixed sources group included participants who obtained foods from at home, restaurant, and fast food sources according to their 24-h recalls. The investigation included no other combinations of food sources. To understand the general intake changes throughout the analysis, we established the overall sources group to include every individual in the sample population regardless of food sources. Sample characteristics are in Additional file 1: Tables S1 and S2.

### Measure of diet quality

We used the HEI-2015 to assess a participant’s diet quality. This method measures specific nutritional elements scored on a density basis (i.e., per 1000 kcals). The HEI-2015 is divided into 13 components that have a maximum score of either 5 or 10 depending on the item. These components then sum together for a maximum total score of 100 [39]. We selected the HEI-2015 because it provides both a component score that encompasses the major goals in the 2015–2020 Dietary Guidelines for Americans and a total score that allows researchers to compare overall diet quality patterns across time and socioeconomic strata [38].

We used the Food Patterns Equivalents Database (FPED) [40], which groups food items based on their nutritional components, to calculate HEI-2015 scores. In the NHANES 2011–2014 data the food items were already classified in such a way that they were readily converted to the FPED and easily run through the HEI-2015 macro. The CSFII 1989–1991 data, on the contrary, required a more complex process [41]. First, we converted food items from the CSFII 1989–1991 to the CSFII 1994–1996 data set, as this newer data set has a connection to the MyPyramid Equivalents Database version 1.0 (MPED 1) that the older data set does not have [32, 42]. We reorganized food items from the CSFII 1989–1991 with food codes identical or extremely similar to the CSFII 1994–1996 and successfully converted them to the CSFII 1994–1996 data set and thereafter to the MPED 1 [41]. However, some food items did not convert perfectly. These foods, spread out across 11% of the sample population, resulted in incomplete dietary information for these individuals, as these foods went unrecognized in the MPED 1. Since these participants did not differ in demographic or macronutrient composition from the main sample, we excluded them from the study. After we converted the data to the MPED 1, we easily converted them to the more comprehensive FPED and subsequently ran them through the HEI-2015 macro to generate HEI scores.

### Statistical analysis

During this investigation we used nationally weighted data. We used the population ratio method to better reflect usual intake at the group level by generating HEI-2015 scores as outlined by the National Cancer Institute [43–45]. This approach allowed us to investigate the quality of foods on days in which food sources were used versus not, and assess HEI-2015 differences between the two selected time points. All analyses were run through the software program SAS version 9.3 (SAS Institute) and were unadjusted [46]. The official population ratio method HEI-2015 macro creates a ratio using the population’s total inake of a particular food group relevant to the HEI-2015 component scores and the population’s total energy intake. Based on these ratios, the macro is then able to calculate each of the HEI-2015 component scores [43–45]. Since there is no documented testing protocol for determining statistical significance between two different HEI scores while using the population ratio method [43, 44], we made comparisons based on the calculated 95% confidence intervals. Specifically, if the confidence intervals of two different results did not overlap, it was determined that these results were significantly different. Conversely if the confidence intervals did overlap, they were determined to not be statistically different. This approach has been reported in other investigations using the population ratio method to calculate HEI-2015 scores [47].

## Results

### Changes in diet quality

Using HEI-2015 component and total scores, Table 1 presents the detailed breakdown of changes in diet quality between 1989 and 1991 and 2011–2014. The HEI score for the entire sample increased by 7.1. This overall increase in diet quality was spread across all the food sources, as all source groups showed an increase in HEI scores. The restaurant group had the largest increase in HEI-2015 score, 14.6, while the fast food group had the smallest increase, 5.9, on days it was consumed. The fast food group remained the least healthy of the four food source groups with a mean score of 51.4 in 2011–2014, whereas the restaurant group overtook the at home group as the healthiest with HEI scores of 63.0 (95% CI: 60.2, 65.6) and 61.5 (59.0, 63.8), respectively.

**Table 1:** HEI-2015 Scores for Young Adults (18–39 years) by Food Source Group, CSFII 1989–1991 and NHANES 2011–2014^a^

	CSFII 1989–1991					NHANES 2011–2014				
HEI Components (95% Confidence Interval)	At Home^b^ (*n* = 2255)	Restaurant^b^ (*n* = 385)	Fast Food^b^ (*n* = 1355)	Mixed Sources^b^ (*n* = 222)	Overall Sources^b^	Change in At Home (*n* = 971)	Change in Restaurant (*n* = 408)	Change in Fast Food (*n* = 1793)	Change in Mixed Sources (*n* = 623)	Change in Overall Sources
Total Fruits	2.7	2.7	2.1^c^	1.8^c^	2.4	0.5^d^	0.7	0.0^c^	0.4^c^	0.1
maximum score: 5	(2.4, 3.0)	(2.2, 3.2)	(1.9, 2.4)	(1.4, 2.2)	(2.2, 2.6)	(3.0, 3.4)	(2.6, 4.2)	(1.9, 2.3)	(1.9, 2.6)	(2.4, 2.7)
Whole Fruits	3.0	2.7	2.0^c^	1.4^c^	2.5	1.5^d^	1.8^d^	0.5^c, d^	1.5^c, d^	0.8^d^
maximum score: 5	(2.5, 3.4)	(2.0, 3.4)	(1.8, 2.3)	(0.9, 1.8)	(2.2, 2.7)	(4.0, 5.0)	(3.4, 5.0)	(2.3, 2.8)	(2.2, 3.5)	(3.0, 3.5)
Total Vegetables	3.2	3.9^c^	3.1	3.6	3.3	−0.3^d^	−0.5^c, d^	− 0.6^c, d^	−0.6^d^	− 0.5^d^
maximum score: 5	(3.1, 3.4)	(3.7, 4.2)	(2.9, 3.3)	(3.3, 4.0)	(3.2, 3.4)	(2.7, 3.1)	(3.1, 3.7)	(2.4, 2.7)	(2.8, 3.3)	(2.7, 2.9)
Greens & Beans	2.3	1.5	1.5^c^	1.9	1.9	2.1^d^	2.0^c, d^	0.6^c, d^	1.1^c, d^	1.1^d^
maximum score: 5	(1.9, 2.7)	(0.9, 2.0)	(1.2, 1.8)	(1.3, 2.5)	(1.6, 2.1)	(3.9, 5.0)	(3.0, 3.9)	(1.8, 2.3)	(2.5, 3.5)	(2.7, 3.2)
Whole Grains	2.2	1.3^c^	1.6^c^	1.5^c^	1.8	1.0^d^	1.9^d^	0.5^c, d^	0.4^c^	0.6^d^
maximum score: 10	(1.9, 2.5)	(1.0, 1.6)	(1.3, 1.9)	(1.0, 1.9)	(1.6, 2.0)	(2.8, 3.6)	(2.5, 4.0)	(1.9, 2.4)	(1.5, 2.3)	(2.2, 2.7)
Dairy	6.6	5.7	6.3	4.8^c^	6.2	0.1	−0.5^c^	−0.3	1.2^d^	−0.1
maximum score: 10	(6.1, 7.1)	(5.0, 6.5)	(5.7, 6.9)	(4.2, 5.4)	(5.9, 6.6)	(6.1, 7.3)	(4.7, 5.8)	(5.6, 6.4)	(5.5, 6.5)	(5.8, 6.4)
Total Protein Foods	5.0	5.0	5.0	5.0	5.0	0.0	0.0	0.0	0.0	0.0
maximum score: 5	(5.0, 5.0)	(5.0, 5.0)	(4.9, 5.0)	(4.9, 5.0)	(5.0, 5.0)	(5.0, 5.0)	(5.0, 5.0)	(5.0, 5.0)	(5.0, 5.0)	(5.0, 5.0)
Seafood and Plant Proteins	3.0	3.6	2.5	2.6	2.8	1.6^d^	1.3^d^	0.4^c^	1.4^d^	0.9^d^
maximum score: 5	(2.5, 3.5)	(3.0, 4.2)	(2.1, 3.0)	(2.0, 3.1)	(2.5, 3.2)	(3.8, 5.0)	(4.2, 5.0)	(2.4, 3.6)	(3.2, 5.0)	(3.3, 4.2)
Fatty Acids	3.2	2.9	3.0	3.7	3.1	1.2^d^	2.6^c, d^	1.5^d^	1.1^d^	1.5^d^
maximum score: 10	(2.9, 3.6)	(2.4, 3.5)	(2.5, 3.5)	(3.2, 4.4)	(2.9, 3.4)	(4.0, 4.8)	(4.9, 6.2)	(4.0, 5.0)	(4.4, 5.4)	(4.3, 5.0)
Refined Grains	5.4	5.8	4.7	4.6	5.1	0.7^d^	1.4^c, d^	0.8^d^	1.7^d^	0.8^d^
maximum score: 10	(5.2, 5.6)	(5.0, 6.5)	(4.2, 5.2)	(3.5, 5.7)	(4.8, 5.5)	(5.6, 6.4)	(6.5, 7.8)	(5.2, 5.8)	(5.8, 6.8)	(5.7, 6.2)
Sodium	3.8	2.8	3.9	4.7	3.8	0.2	0.1	0.6	−1.0	0.3
maximum score: 10	(3.4, 4.1)	(1.8, 3.8)	(3.4, 4.5)	(3.8, 5.7)	(3.5, 4.1)	(3.3, 4.6)	(2.3, 3.6)	(4.3, 4.8)	(3.2, 4.2)	(3.8, 4.3)
Added Sugars	7.0	6.9	5.7^c^	5.8^c^	6.4	−0.4^d^	0.2^c^	−0.3^c^	0.4	−0.4^d^
maximum score: 10	(6.8, 7.2)	(6.2, 7.6)	(5.3, 6.1)	(4.8, 6.8)	(6.2, 6.6)	(6.3, 6.8)	(6.8, 7.4)	(5.0, 5.7)	(5.7, 6.7)	(5.8, 6.2)
Saturated Fats	4.7	3.6^c^	4.0	4.4	4.3	1.3^d^	3.7^c, d^	2.2^d^	1.7^d^	2.0^d^
maximum score: 10	(4.4, 5.1)	(2.8, 4.3)	(3.4, 4.6)	(3.2, 5.6)	(3.9, 4.6)	(5.5, 6.7)	(6.7, 7.7)	(5.8, 6.5)	(5.6, 6.7)	(5.9, 6.5)
Total HEI-2015 Score	52.0	48.4^c^	45.5^c^	45.8^c^	48.7	9.5^d^	14.6^d^	5.9^c, d^	9.4^c, d^	7.1^d^
maximum score: 100	(50.3, 53.8)	(46.7, 50.1)	(44.1, 47.0)	(43.3, 48.5)	(47.6, 49.8)	(59.0, 63.8)	(60.2, 65.6)	(49.7, 53.2)	(52.8, 57.6)	(54.6, 57.0)

^a^Data in this table are nationally weighted and unadjusted

^b^At home refers to those who consumed food strictly from at home sources for the 24-h recall period. Restaurant refers to those who ate food from a restaurant and an at home source. Fast food refers to those who ate food from a fast food and an at home source. Mixed sources refers to those who ate food from all three other sources, restaurant, fast food, and at home. Overall sources refers to the entire sample regardless of food source

^c^Significantly (*P* < 0.05) different from the at home food source

^d^ Significantly (*P* < 0.05) different between 1989 and 1991 and 2011–2014

In regard to results from the specific HEI-2015 component scores, the total vegetables score decreased across all food source groups. However, the greens and beans category increased across all sources, and the at home group had a significantly higher score, 4.4 (3.9, 5.0), compared to the rest of the food source groups. Overall the added sugars component score decreased significantly by 0.4 in the HEI-2015 score, which means the young adult population consumed more added sugars in 2011–2014 than in 1989–1991. Furthermore, dairy consumption did not experience relevant changes over the years, as only the mixed sources group showed a significant increase in dairy consumption. The overall increase in HEI total scores between 1989 and 1991 and 2011–2014 was largely accredited to the whole fruits, fatty acids, refined grains, and saturated fats component scores, as these significantly increased across all four groups. Moreover, whole grains and seafood and plant proteins scores increased significantly across three of the four groups.

### Socioeconomic changes and disparities

**Fig. 1:**
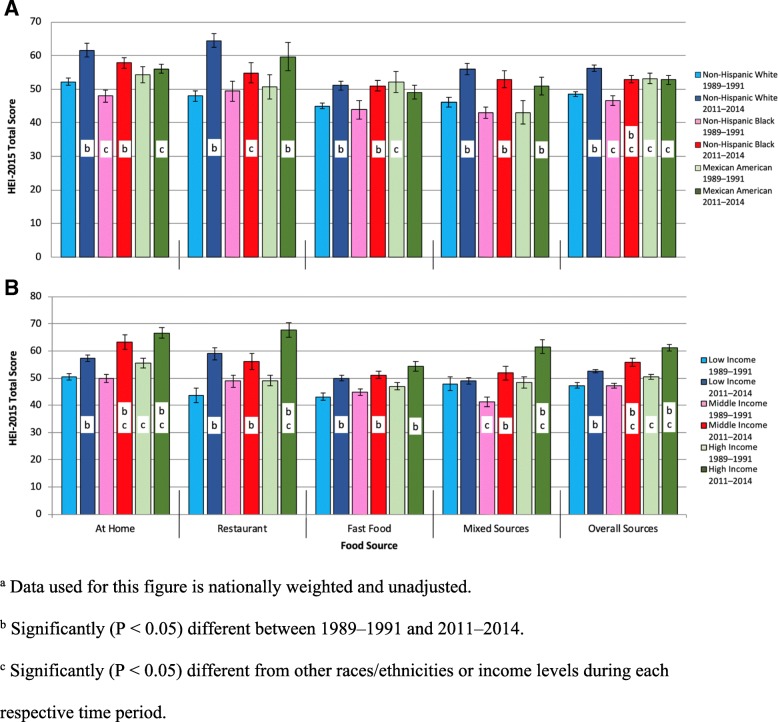
Changes in HEI Scores by Socioeconomic Variable and Food Source between 1989 and 1991 and 2011–2014^a^. ^a^ Data used for this figure is nationally weighted and unadjusted. ^b^ Significantly (*P* < 0.05) different between 1989 and 1991 and 2011–2014. ^c^ Significantly (*P* < 0.05) different from other races/ethnicities or income levels during each respective time period. **a** shows changes in HEI-2015 scores from 1989-1991 to 2011-2014 based on food source and race/ethnicity. **b** presents changes in HEI-2015 scores based on food source and family income

As illustrated in Fig. 1a, in the overall sources category NHWs and NHBs showed significant increases in HEI scores, whereas Mexican Americans showed no change over this 23-year period (see Additional file 1: Table S3 for detailed HEI score information). Although in 1989–1991 Mexican Americans had the best diet quality of the three race/ethnicity groups, by 2011–2014 the NHWs had the highest HEI scores, showing a race/ethnic disparity in the consumption of nutritious foods. With the exception of the restaurant group, where the NHWs showed a significant increase of 16.5 points in HEI scores, NHWs and NHBs experienced a similar degree of improvement in the other food sources. Though not statistically significant, Mexican Americans only experienced a decrease in the fast food group, where they had a decrease of 3.0 points.

Regarding income, Fig. 1b shows an increase in HEI scores among all income categories (see Additional file 1: Table S4 for more detailed HEI score information). Even though all income categories improved their HEI scores, there was a disproportionate increase in HEI score depending on the income level. Specifically, the greater the income level, the greater the observed improvement in the overall HEI score. This trend is supported by the fact that the low-income group increased its HEI score by 5.1, the middle-income group increased by 8.7, and the high-income group increased by 10.7 over the time period. The high-income group also experienced higher scores throughout all the food sources both in 1991 and in 2014. Moreover, while the low- and middle-income groups had similar overall HEI scores of approximately 47.2 in 1991, greater growth in the at home and mixed source categories over the two decades allowed the middle-income group to separate itself from the low-income group. In addition, the mixed source category showed the largest socioeconomic disparity, as the middle- and high-income groups showed significant increases in HEI scores, while the low-income group showed no improvement. Finally on days in which fast food was consumed, the fast food category represented the smallest difference among the income levels, as each of the three groups saw an improvement of around 7.0, with the high-income group increasing by only 0.5 points more than the low-income group.

## Discussion

Results from this study reveal that between 1989 and 1991 and 2011–2014 young adult diet quality measured through HEI-2015 scores improved across all food sources. Despite this improvement in overall scores, both the total vegetables and the added sugars component scores decreased during this time period. Although NHWs and NHBs both saw increases in diet quality across food sources, Mexican Americans saw no changes. Furthermore, all income levels experienced increases in diet quality. However, a widening nutritional disparity is emerging as the high-income group experienced greater improvements in diet quality compared to the low- and middle-income groups.

The most remarkable change in the specific HEI component scores was the decrease in total vegetables across all four sources. This finding is of concern, because decreased vegetable consumption has been linked to increased risk of multiple chronic diseases [48, 49]. Furthermore, the HEI score for added sugars also worsened over the 23-year period, suggesting that young adults in the United States now consume more calories from added sugars than in years prior. This overall decrease in score is largely accredited to at home sources, as no other food source saw a significant change in added sugars. It is imperative to note that other research has revealed that added sugar intake has declined significantly since 2002–2003 due mainly to shifts away from sugar-sweetened beverages [11, 35]. Nevertheless, the increase between 1989 and 1991 and 2011–2014 is problematic and provides perspective on the longer-term changes in added sugar intake. While the 2015–2020 Dietary Guidelines encourage Americans to consume less than 10% of their daily calories from added sugars, a recommendation with which the World Health Organization agrees, stronger action is required, as adults still obtain over 13% of their calories from added sugars due largely to purchases of processed foods high in sugar [38, 50–52].

The discrepancy in nutritional improvements based on racial/ethnic differences is also of concern. Notably, Mexican Americans experienced no change in diet quality between 1991 and 2014, while NHWs and NHBs experienced major increases. NHWs and NHBs experienced similar absolute increases across the at home, fast food, and mixed sources groups. However, NHWs saw a much greater increase in restaurant sources compared to NHBs, on days in which restaurant food was consumed. In regard to Mexican Americans, lack of changes in the at home and fast food groups, the two largest in this study, are responsible for the observed stagnation in the overall HEI score for this demographic group over the time period. One possible explanation for this stagnant development is food acculturation as immigrants adopt unhealthy eating habits in the United States [53, 54]. This theory is supported by other studies that show that in Mexico, Mexicans eat a healthier and more traditional diet compared to their immigrant counterparts in the United States [53–56]. Since the influence of traditional Mexican diets can be lost after only one generation in the United States, efforts to protect the beneficial attributes of Mexican diets while discouraging adoption of unhealthy American habits need to be considered [53].

Income level also influences diet quality. Although all HEI-2015 scores improved for each income group, the absolute and relative disparities between low- and high-income groups increased. Interestingly, all income groups experienced similar absolute increases in the fast food source, which exhibits the smallest difference in HEI-2015 scores between low- and high-income levels. The income disparity in overall sources is therefore largely due to substantial increases for high-income individuals in the restaurant and mixed sources. One potential explanation of this separation is that low-income individuals are more likely to consume processed, energy-dense foods than high-income individuals [57–59]. This is directly linked to the relatively cheap prices of unhealthy foods and low-income individuals’ limited access to healthy food sources [59–61]. Moreover, people with low-incomes have fewer choices in available types and sources of food than middle- and high-income groups [62, 63]. As suggested by other studies, efforts to improve the nutritional quality of low-income individuals should target the accessibility and affordability of healthy foods in store, restaurant, and fast food sources to limit the disparity in nutritional quality among different economic strata [60, 61].

We focused on young adults ages 18 to 39 for a number of reasons. First, few studies focus on this specific demographic, as traditional diet quality studies tend to investigate children or adults in general. Gaining knowledge on young adults in particular is valuable, because young adults are the most likely age group to eat out and also are heavy consumers of sugary beverages and snacks [7, 20, 24, 64–66]. Additionally, studying young adults is crucial because they are more likely to be raising a family. Numerous studies have determined that parents play an instrumental role in shaping children’s eating habits that will persist throughout a majority of their lives; therefore, it is imperative to understand the dietary choices parents make for themselves and likely passed on to their children [67–69]. Finally, young adulthood is a period of critical weight gain and consequently requires specific attention [31].

HEI-2015 as a measure of diet quality is a powerful tool to better understand health patterns in the United States. Many other studies show an inverse relationship between HEI scores and risk of chronic diseases and overall mortality [70–73]. This study, however, revealed that diet quality has improved over the same time period that obesity rates have increased in the US [1–4]. One explanation for this disconnect might be the fact HEI scores are based on a nutrient density measure that does not take into consideration the amount of total calories consumed [39]. Since the HEI-2015 only analyzes the types of calories eaten, the scores ignore the problems associated with excess total calories consumed and positive energy balances. Another explanation for this disconnect could be that even though diet quality did improve, in absolute terms the diet quality is still relatively low.

This study has limitations. First, we included only two time points to examine changes in HEI-2015 scores over 23 years, which hindered our ability to report any detailed trends that occurred during this time period. For example, the consumption of sugar-sweetened beverages and foods peaked in 2002–2003 [11, 35], so this study might have missed the highest period of added sugar consumption by concentrating on 1989–1991 and 2011–2014. Although using additional years of NHANES data would have shed additional light on the trends in diet quality based on food source, the aim of this paper was not to address exhaustively every change that occurred for each food component score from time period to time period. Rather by analyzing overall changes over this 23-year span, we were able to focus on major long-term diet quality issues, such as the diet disparities that are emerging due to socioeconomic differences, and explore why some of these issues may exist today.

Another limitation is that we lost 11% of the sample due to analytic difficulties in the creation of the 1989–1991 HEI. However, sensitivity analyses showed no differences between the included and excluded populations, so the effects of this exclusion should be minimal. An additional limitation is that the investigation did not address possible changes in the food industry and behavioral attitudes toward food sources. Selectivity in food service type is an important variable to consider, as it could potentially affect the results. However, one study found that fast food consumers eat the same way at home and at a fast food restaurant, so the nutritional impact of this food source is a lesser issue compared to the overall dietary pattern [29]. In a further limitation, insufficient information is available on dishes and preparations for the large variety of foods in the restaurant category of the away from home sources. The lack of food composition tables with accurate information on restaurant foods could cause misrepresentation of the diet quality of foods from these sources in our study. No existing database can help resolve this problem. Another limitation is that two cells in the socioeconomic analysis, the 1989–1991 mixed sources category for NHBs and Mexican Americans, contained fewer than 40 individuals. Thus results for these specific groups should be examined with caution given the insufficient sample size.

An additional limitation is that the nature of fast food has evolved over time, as fast food chains have changed to include healthier options, such as salads, and modified versions of main entrees to become healthier (e.g. baked vs fried). Although we cannot measure or control for these supply changes, with the inclusion of these healthier options we would expect that the fast-food group experience would be linked to greater increases in their HEI scores compared to if no changes in the fast food indstury had occur. That is if fast food eaters now include salad and other healthier eaters, the nonfastfood component of the day would be expected to be healthier. However, our findings show that on days it was consumed, fast food remained the lowest HEI score with the least improvement, thus, this limitation actually strengthens our conclusion. In further limitation, we understand that using a single dietary recall does not accurately capture consumers usual dietary habits. However, our aim was not to asses usual diet based on the probability of using food sources on multiple days, but rather asses the diet quality from food sources on a single given day. Furthermore, the second day of data was not comparable between surveys, and classifying individuals into consumers or not of a food source at a given day using both days would be impossible, as one day food sources might differ form the other. A final limitation is that underreporting of fast food may have increased over time given social desirability biases related to stigma associated with unhealthy foods and with overweight and obesity.

## Conclusion

This study reveals that diet quality measured through HEI-2015 scores improved across all food sources. Despite this improvement, socioeconomic disparities have emerged, as Mexican Americans and low-income individuals saw little or no improvements in HEI scores over the 23-year period. Future studies should consider evaluating total calorie intake in addition to diet quality. Additionally, concentrated efforts are needed to improve the diet quality of the high-risk demographic groups identified. Finally, future work should attempt to identify how certain food sources influence the remainder of an individual’s diet while also investigating why specific food sources contribute more to nutritional disparities based on socioeconomic status than others.

## Additional file

Additional file 1: **Table S1.** Distribution of a cohort of 8012 young adults (ages 18–39) based on socioeconomic characteristics and the combination of sources they obtained their food from during a 24-h dietary recall. **Table S2.** Distribution of a cohort of 8012 young adults (ages 18–39) by their socioeconomic variables stratified by the time period and the combination of sources they obtained their food from during a 24-h dietary recall. **Table S3.** HEI-2015 Scores for Young Adults (18–39) by Food Source and Race/Ethnicity, from CSFII 1989**–**91 and NHANES 2011**–**14^a^. **Table S4.** HEI-2015 Scores for Young Adults (18–39) by Food Source and Income, from CSFII 1989**–**91 and NHANES 2011**–**14^a^. (DOCX 92 kb)

## Data Availability

This investigation used three data sets: (1) Continuing Survey of Food Intakes by Individuals 1989–1991, US Department of Agriculture, https://www.ars.usda.gov/northeast-area/beltsville-md-bhnrc/beltsville-human-nutrition-research-center/food-surveys-research-group/docs/csfii-1989-1991-and-dhks-1989-1991/ (2) National Health and Nutrition Examination Survey 2011–2012, Centers for Disease Control and Prevention, https://wwwn.cdc.gov/nchs/nhanes/ContinuousNhanes/Default.aspx?BeginYear=2011 (3) National Health and Nutrition Examination Survey 2013–2014, Centers for Disease Control and Prevention, https://wwwn.https://wwwn.cdc.gov/nchs/nhanes/continuousnhanes/default.aspx?BeginYear=2013.
