# Supplementary material for: Improvements in the nutritional quality of US young adults based on food sources and socioeconomic status between 1989–1991 and 2011–2014
Source: Nutr J. 2019 Jun 26;18:32. doi: 10.1186/s12937-019-0460-4 (PMC6595624; doi:10.1186/s12937-019-0460-4)
Supplement: Supplementary file 1 — Table S1. Distribution of a cohort of 8012 young adults (ages 18–39) based on socioeconomic characteristics and the combination of sources they obtained their food from during a 24-h dietary recall. Table S2. Distribution of a cohort of 8012 young adults (ages 18–39) by their socioeconomic variables stratified by the time period and the combination of sources they obtained their food from during a 24-h dietary recall. Table S3. HEI-2015 Scores for Young Adults (18–39) by Food Source and Race/Ethnicity, from CSFII 1989–91 and NHANES 2011–14a. Table S4. HEI-2015 Scores for Young Adults (18–39) by Food Source and Income, from CSFII 1989–91 and NHANES 2011–14a. (DOCX 92 kb) [file 12937_2019_460_MOESM1_ESM.docx]

**Supplemental Material:**

| **Supplemental Table 1**. Distribution of a cohort of 8,012 young adults (ages 18-39) based on socioeconomic characteristics and the combination of sources they obtained their food from during a 24-hour dietary recall | | |
| --- | --- | --- |
| **Variable:** | **CSFII 1989–1991^a^** | **NHANES 2011–2014^a^** |
| Total Sample Population: | N=4217 | N=3795 |
| **Food Source:** |  |  |
| At- Home, (%) | 53.5 | 25.6 |
| Restaurant, (%) | 9.1 | 10.8 |
| Fast Food, (%) | 32.1 | 47.2 |
| Mixed Sources, (%) | 5.3 | 16.4 |
| **Race and Origin:** |  |  |
| Non-Hispanic White, (%) | 71.4 | 37.0 |
| Non-Hispanic Black, (%) | 13.8 | 21.7 |
| Mexican American, (%) | 11.2 | 14.2 |
| **Income Status:** |  |  |
| Low Income, (%) | 54.7 | 53.2 |
| Middle Income, (%) | 25.0 | 21.2 |
| High Income, (%) | 20.3 | 24.8 |

^a^ Data used for this table is nationally weighted and unadjusted.

| **Supplemental Table 2**. Distribution of a cohort of 8,012 young adults (ages 18-39) by their socioeconomic variables stratified by the time period and the combination of sources they obtained their food from during a 24-hour dietary recall | | | | | | | | | |  |
| --- | --- | --- | --- | --- | --- | --- | --- | --- | --- | --- |
|  | **1989–1991 Continuing Survey of Food Intakes by Individuals^a^** | | | |  | **2011–2014 National Health and Nutrition Examination Survey^a^** | | | | |
|  | Food Source: | | | |  | Food Source: | | | | |
|  | At Home | Restaurant | Fast-Food | Mixed Sources |  | At Home | Restaurant | Fast-Food | Mixed Sources | |
| Sociodemographic Variable: |  |  |  |  |  |  |  |  |  | |
| **Race/Ethnicity** |  |  |  |  |  |  |  |  |  | |
| Non-Hispanic White, (%) | 49.9 | 9.7 | 34.3 | 6.1 |  | 26.4 | 10.2 | 46.7 | 16.7 | |
| Non-Hispanic Black, (%) | 61.0 | 7.0 | 29.5 | 2.6 |  | 20.9 | 6.8 | 57.3 | 15.0 | |
| Mexican American, (%) | 62.8 | 8.9 | 25.5 | 2.8 |  | 26.8 | 11.7 | 48.8 | 12.7 | |
| **Income Status** |  |  |  |  |  |  |  |  |  | |
| Low Income, (%) | 61.0 | 6.6 | 29.5 | 2.9 |  | 27.9 | 7.5 | 51.6 | 13.0 | |
| Middle Income, (%) | 48.5 | 10.4 | 34.8 | 6.3 |  | 24.5 | 11.1 | 46.8 | 17.6 | |
| High Income, (%) | 39.4 | 14.3 | 36.0 | 10.3 |  | 20.8 | 16.9 | 39.2 | 23.2 | |

^a^ Data used for this table is nationally weighted and unadjusted.

| **Supplemental Table 3.** HEI-2015 Scores for Young Adults (18-39) by Food Source and Race/Ethnicity, from CSFII 1989**–**91 and NHANES 2011**–**14^a^ | | | | | | | | | | | | | |  |
| --- | --- | --- | --- | --- | --- | --- | --- | --- | --- | --- | --- | --- | --- | --- |
|  | **1989–1991 Continuing Survey of Food Intakes by Individuals** | | | | | |  | **2011–2014 National Health and Nutrition Examination Survey** | | | | | | |
| Race/Ethnicity | Sample Size | At Home | Restaurant | Fast Food | Mixed Sources | Overall Sources |  | Sample Size | At Home | Restaurant | Fast Food | Mixed Sources | Overall Sources | |
| **Non-Hispanic White** | 3010 | 52.2 | 48.0 | 45.0 | 46.1 | 48.4 |  | 1404 | 61.6^b^ | 64.5^b^ | 51.1^b^ | 56.0^b^ | 56.2^b^ | |
| *SD* |  | *50.5-54.0* | *44.8-51.2* | *43.5-46.5* | *43.2-49.1* | *47.7-49.2* |  |  | *57.8-65.2* | *60.5-68.2* | *48.3-53.9* | *52.9-59.3* | *54.8-57.7* | |
| **Non-Hispanic Black** | 584 | 48.0^c^ | 49.4 | 43.9 | 43.0^d^ | 46.7^c^ |  | 824 | 57.8^b^ | 54.9^c^ | 51.0^b^ | 52.9^b^ | 52.9^b,c^ | |
| *SD* |  | *45.8-50.4* | *43.1-55.0* | *39.8-47.9* | *39.4-46.2* | *45.6-47.7* |  |  | *54.8-60.6* | *50.4-60.0* | *48.2-53.5* | *47.6-57.4* | *51.1-54.7* | |
| **Mexican American** | 471 | 54.3 | 50.7 | 52.1^c^ | 43.1^d^ | 53.2^c^ |  | 537 | 56.1^c^ | 59.7^b^ | 49.1 | 50.9^b^ | 52.8^c^ | |
| *SD* |  | *49.8-58.6* | *45.5-55.7* | *47.0-57.1* | *39.9-46.2* | *50.0-56.4* |  |  | *54.3-57.7* | *55.8-63.6* | *44.9-53.1* | *46.6-55.2* | *51.2-54.4* | |

^a^ Data used for this table is nationally weighted and unadjusted.

^b^ Significantly (P < 0.05) different between 1989**–**91 and 2011**–**2014.

^c^ Significantly (P < 0.05) different from Non-Hispanic White.

^d^ Data in these cells contain an insignificant sample size (n < 40).

| **Supplemental Table 4.** HEI-2015 Scores for Young Adults (18-39) by Food Source and Income, from CSFII 1989**–**91 and NHANES 2011**–**14^a^ | | | | | | | | | | | | | |  |
| --- | --- | --- | --- | --- | --- | --- | --- | --- | --- | --- | --- | --- | --- | --- |
|  | **1989–1991 Continuing Survey of Food Intakes by Individuals** | | | | | |  | **2011–2014 National Health and Nutrition Examination Survey** | | | | | | |
| Income Status | Sample Size | At Home | Restaurant | Fast Food | Mixed Sources | Overall Sources |  | Sample Size | At Home | Restaurant | Fast Food | Mixed Sources | Overall Sources | |
| **Low Income** | 2306 | 50.5 | 43.7 | 43.1 | 47.9 | 47.3 |  | 2020 | 57.2^b^ | 59.0^b^ | 50.0^b^ | 49.1 | 52.4^b^ | |
| *SD* |  | *48.3-52.7* | *38.5-49.3* | *40.5-45.8* | *43.5-52.1* | *45.8-48.8* |  |  | *54.8-59.6* | *55.0-62.6* | *47.9-52.0* | *46.8-51.6* | *51.2-53.6* | |
| **Middle Income** | 1053 | 49.9 | 48.9 | 44.7 | 41.3^c^ | 47.1 |  | 806 | 63.2^b,c^ | 56.0^b^ | 51.2^b^ | 51.8^b^ | 55.8^b,c^ | |
| *SD* |  | *47.1-52.8* | *46.1-51.9* | *42.4-47.0* | *39.1-43.4* | *45.6-48.8* |  |  | *59.9-66.5* | *52.0-59.5* | *48.6-53.6* | *46.7-56.5* | *53.8-57.7* | |
| **High Income** | 858 | 55.4^c^ | 49.1 | 47.0 | 48.4 | 50.5^c^ |  | 941 | 66.6^b,c^ | 67.7^b,c^ | 54.4^b^ | 61.6^b,c^ | 61.2^b,c^ | |
| *SD* |  | *52.8-57.9* | *45.2-52.7* | *44.2-49.8* | *44.2-52.9* | *49.0-52.0* |  |  | *62.4-70.0* | *62.9-72.1* | *51.0-57.9* | *56.8-66.3* | *58.9-63.6* | |

^a^ Data used for this table is nationally weighted and unadjusted.

^b^ Significantly (P < 0.05) different between 1989**–**91 and 2011**–**2014.

^c^ Significantly (P < 0.05) different from Low Income.
